# Real-World Treatment Patterns and Vision Outcomes with Ranibizumab for Diabetic Macular Edema

**DOI:** 10.1155/2021/8825082

**Published:** 2021-01-27

**Authors:** Tadas Naujokaitis, Vilma Jurate Balciuniene

**Affiliations:** ^1^Faculty of Medicine, Lithuanian University of Health Sciences, Kaunas, Lithuania; ^2^Department of Ophthalmology, Hospital of the Lithuanian University of Health Sciences Kauno Klinikos, Kaunas, Lithuania

## Abstract

**Purpose:**

To assess injection patterns and vision outcomes in patients receiving intravitreal ranibizumab injections for diabetic macular edema in a real-world clinical setting.

**Methods:**

Retrospective chart review involving 74 eyes of 62 patients who started ranibizumab treatment for diabetic macular edema at the Hospital of the Lithuanian University of Health Sciences Kauno Klinikos. Data collected included follow-up visits, injections administered, and best-corrected visual acuity (BCVA).

**Results:**

Median follow-up duration was 652.5 days (min 365; max 914). Over the first year, eyes received a median of 4 injections (min 1; max 10). Among eyes with 2-year follow-up and injections during the second year, there was a median of 3 injections (min 1; max 6) over the second year. The BCVA improved by a median of 5 letters 365 ± 60 days and 730 ± 60 days after baseline. At the first visit ≥365 days after baseline, 13.5% of eyes gained ≥15 letters from baseline while 6.8% of eyes lost ≥15 letters. For 74.3% of eyes, BCVA improved (gain of ≥5 letters) or remained stable (gain/loss of ≤4 letters).

**Conclusion:**

Intravitreal ranibizumab for diabetic macular edema was effective in a real-world clinical setting, with most eyes gaining or maintaining vision. Compared with randomized prospective clinical trials, patients received less frequent injections and achieved lower vision gains.

## 1. Introduction

Diabetic macular edema (DME) affects more than 20 million people worldwide and causes most of the vision loss among patients with diabetes [1, 2]. Following the discovery of the role of vascular endothelial growth factor in the pathogenesis of DME, antivascular endothelial growth factor (anti-VEGF) agents were created. In recent years, anti-VEGF intravitreal injections have become the mainstay of DME treatment and have largely replaced the macular laser photocoagulation [3]. Future treatment approaches in patients with diabetic retinopathy may include not only anti-VEGF agents but also human stem cells. Intravitreal injections of human mesenchymal stem cells, as well as CD34+ bone marrow stem cells, have shown promising results in animal models [4, 5]. However, further investigation is required to evaluate the feasibility of such treatment approaches in humans.

Two anti-VEGF agents are currently approved for the treatment of DME: ranibizumab (Lucentis®, Genentech, Inc., South San Francisco, CA, USA) and aflibercept (Eylea®, Regeneron Pharmaceuticals, Inc., Tarrytown, NY, USA). Off-label use of bevacizumab (Avastin®, Genentech, Inc., South San Francisco, CA, USA) is also common due to its significantly lower price [6–8].

Ranibizumab was the first anti-VEGF drug approved for the treatment of DME [9]. Before that, it has been successfully used in patients with neovascular age-related macular degeneration. In these patients, improvements in best-corrected visual acuity (BCVA), macular sensitivity, and fixation stability have been reported [10].

Several real-world studies on DME treatment with ranibizumab showed worse visual outcomes than those reported in clinical trials [11–22]. However, due to variations in clinical practice among different countries, more studies are needed to evaluate the real-world use of ranibizumab for DME.

We conducted this study to assess the outcomes of ranibizumab treatment for DME in a real-world clinical setting in Lithuania. We analyzed the number of intravitreal injections administered and the time intervals between them, as well as the changes in BCVA.

## 2. Methods

### 2.1. Study Design

In this retrospective monocenter study, we analyzed medical records of type 1 and type 2 diabetes patients who received ranibizumab treatment for DME at the Hospital of the Lithuanian University of Health Sciences Kauno Klinikos. We included the eyes with DME, which received their first ranibizumab injection between June 2015 and May 2017, had available BCVA data at baseline, a total of ≥1 ranibizumab injection, and ≥365 days of follow-up. Exclusion criteria were the following: intravitreal injection of another anti-VEGF agent or steroids within 12 months before the start of ranibizumab treatment or during the follow-up of 365 days, intraocular surgery during the follow-up of 365 days, significant media opacities interfering with visual acuity at baseline or during the follow-up of 365 days, and advanced ocular pathology, other than diabetic retinopathy (DR), at baseline or during the follow-up of 365 days. In cases where any of the abovementioned events occurred >365 days following the start of ranibizumab treatment, we only analyzed the data prior to such event. The study was approved by the Center of Bioethics of the Lithuanian University of Health Sciences (BEC-MF-214) and adhered to the principles of the “Declaration of Helsinki.”

### 2.2. Study Outcomes

Anonymized data regarding patient and ocular characteristics, as well as previous treatment of DR, were collected at baseline. BCVA was assessed using Snellen charts at baseline and at follow-up visits. We converted Snellen BCVA measurements to approximate Early Treatment Diabetic Retinopathy Study (approxETDRS) letter scores using the method described by Gregori et al. [23]. Baseline BCVA intervals of the subgroup analysis (<55 letters, 56–68 letters, >68 letters) were used previously in the OCEAN study [18].

Ranibizumab injections were administered pro re nata at the discretion of the treating physician. We collected anonymized data regarding ranibizumab injections and follow-up visits. For the outcomes at 365 ± 60 days and 730 ± 60 days after baseline, the visit nearest to 365 days and 730 days, respectively, was used. Monthly results were presented in 30-day periods starting the day 15 after baseline, e.g., month 0 (0–14 days after baseline) and month 1 (15–44 days after baseline).

### 2.3. Statistical Analysis

Statistical analysis was performed using IBM SPSS Statistics software, version 25 (International Business Machines Corporation, Armonk, USA). BCVA values were tested for normal distribution using the Shapiro–Wilk test. Due to the lack of normal distribution in BCVA data, we used nonparametric tests in our statistical analysis. BCVA values at 365 ± 60 days and 730 ± 60 days after baseline among the three baseline BCVA subgroups (<55 letters, 56–68 letters, and >68 letters) were compared using the Kruskal–Wallis test. This test was also applied to compare the baseline BCVA subgroups in terms of change in BCVA at 365 ± 60 days and 730 ± 60 days after baseline. The Wilcoxon signed rank test was used to compare BCVA values at baseline with values at 365 ± 60 days and 730 ± 60 days after baseline. Results are expressed as a median with the lowest and the highest value for quantitative variables and as a percentage for categorical variables. A *p* value <0.05 was considered statistically significant.

## 3. Results

### 3.1. Study Population

Out of 122 eyes that met the inclusion criteria, 30 eyes received bevacizumab injection within 12 months before the start of ranibizumab treatment, 4 eyes had mature cataract, and 1 eye had advanced glaucoma. Over the first year of ranibizumab treatment, 3 eyes received bevacizumab injection, 6 eyes underwent intraocular surgery, 3 eyes presented with hemophthalmus, and 1 eye developed secondary cataract. Seventy-four eyes of sixty-two patients were included in the study. Baseline characteristics of patients and study eyes are presented in Table 1.

### 3.2. Follow-up and Treatment

The median follow-up duration was 652.5 days (min 365; max 914), and there were 30 eyes (40.5%) with the follow-up of ≥24 months. Over the first year, a median of 6 visits (min 2; max 15) occurred. Among eyes with 24 months of follow-up, there was a median of 4 visits (min 1; max 9) over the second year.

Over the first year, a median of 4 injections (min 1; max 10) were administered and 25.7% of eyes received 2 injections or less. Among eyes with the follow-up of ≥24 months and injections over the second year (*n* = 18), a median of 3 injections (min 1; max 6) were administered over the second year of treatment.

**Figure 1:**
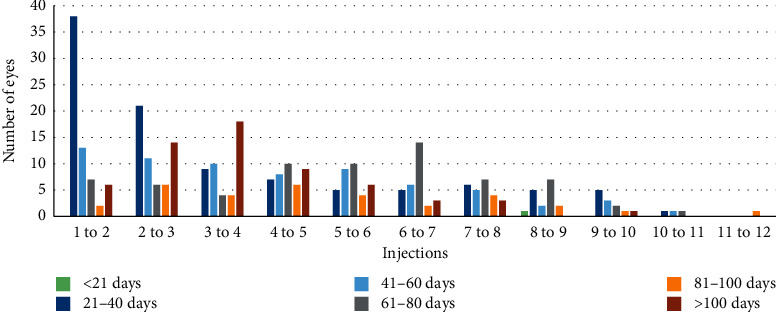
Intervals between injections.

There was a median of 35 days (min 28; max 505) from the first to the second injection, followed by 52.5 days (min 28; max 658) from the second to the third injection. The longest intervals were observed from the third to the fourth injection and from the fourth to the fifth injection.  Figure 1 provides an overview of intervals between injections.

### 3.3. Best-Corrected Visual Acuity

BCVA improved by a median of 5 letters 365 ± 60 days and 730 ± 60 days after baseline, and this change was statistically significant (*p* = 0.026 and *p* = 0.024). BCVA improvement appeared to be greater among eyes with worse baseline BCVA, but there was no statistically significant difference both 365 ± 60 days and 730 ± 60 days after baseline (*p* > 0.05). Eyes with better baseline BCVA (>68 letters) maintained good BCVA throughout the two years of follow-up, and their BCVA remained significantly better than that of eyes with worse baseline BCVA (*p* < 0.05).

**Figure 2:**
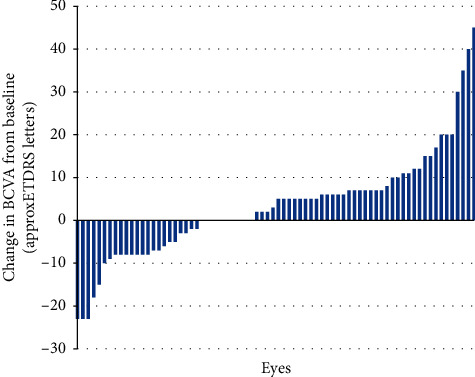
Changes in BCVA from baseline at the first visit ≥365 days after baseline. Data from individual eyes (*n* = 74).

At the first visit ≥365 days after baseline, 13.5% of eyes gained ≥15 letters from baseline while 6.8% of eyes lost ≥15 letters. For 74.3% of the eyes, BCVA improved (gain of ≥5 letters) or remained stable (gain/loss of ≤4 letters). The number of eyes with BCVA of ≥70 letters (≥0.5 Snellen equivalent) increased from 24 eyes (32.4%) at baseline to 33 eyes (44.6%) at the first visit ≥365 days after baseline. BCVA outcomes are presented in Table 2 and Figure 2.

**Figure 3:**
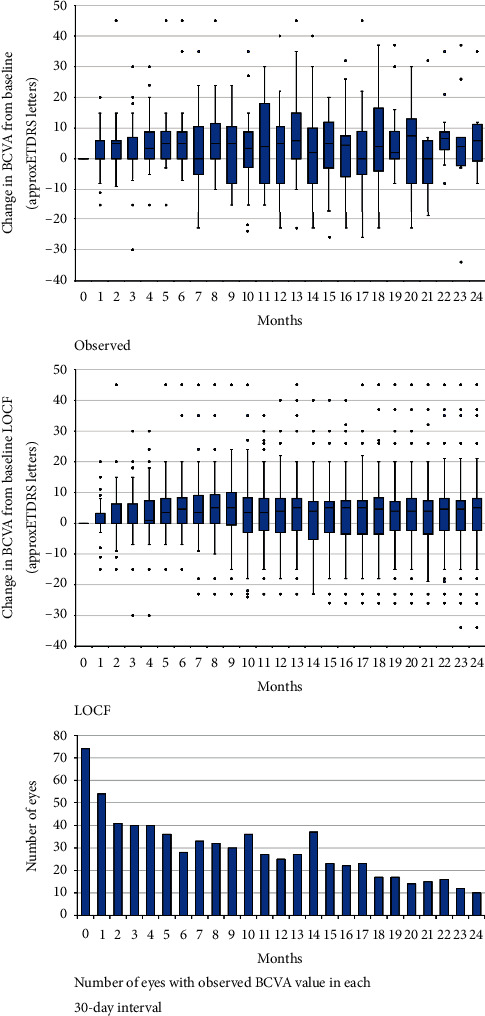
Change in BCVA from baseline over 24 months among all eyes (*n* = 74); observed values only and Last Observation Carried Forward (LOCF) analysis. Quartiles and medians with the lowest and the highest values are shown.

**Figure 4:**
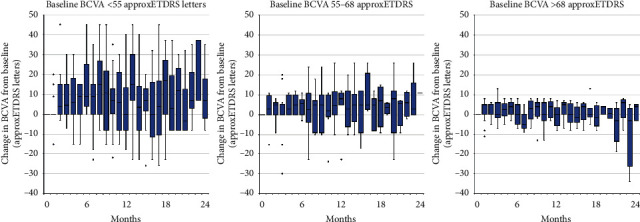
Change in BCVA from baseline over 24 months within baseline BCVA subgroups (observed values only). Quartiles and medians with the lowest and the highest values are shown.

As shown in Figure 3, an improvement in BCVA was observed in the first months following the start of ranibizumab treatment. After that, BCVA remained relatively stable. Last Observation Carried Forward (LOCF) analysis is also presented and includes BCVA data from all study eyes in each 30-day period. Observed changes in BCVA within baseline BCVA subgroups are presented in Figure 4.

## 4. Discussion

DME treatment with ranibizumab is expensive and requires regular follow-up visits. Intensive treatment and monitoring schedules used in clinical trials are difficult to implement in everyday clinical practice, and this could result in different real-world outcomes [24]. Therefore, it is important to evaluate the efficacy of ranibizumab therapy in a real-world clinical setting, where not all patients receive the initial monthly injections and follow-up visits are missed. Furthermore, differences in health systems, resource availability, patient characteristics, and local practice exist among countries, as reported by several recent studies [17–19]. We were aiming to evaluate the outcomes achieved in the largest ophthalmology clinic in Lithuania.

There are clear differences in the treatment intensity between clinical trials and everyday clinical practice. RISE and RIDE clinical trials featured monthly injections, while an average of ≥7 injections were administered over the first year in other clinical trials [11–16]. The median number of injections over the first year of treatment was much lower in our study (4 injections). In addition, some patients did not receive the three loading doses at the start of the treatment. Other real-world studies also reported low numbers of ranibizumab injections. There was an average of 3 to 6 injections over the first year in studies which did not exclude eyes based on the number of injections [17–19, 21].

Real-world studies report not only fewer injections but also usually lower vision gains. After one year of treatment, an average improvement of 5.7–12.5 letters was observed in clinical trials, while it ranged from −1.3 to +6.6 letters in real-world studies [11–22, 24, 25]. We observed the vision gains (+5 letters) that were comparable to those reported in other real-world studies. It should be noted, however, that baseline visual acuity needs to be considered when comparing outcomes of different studies because eyes with worse visual acuity at baseline experience greater vision gains [17, 26]. For example, although 6.6-letter improvement reported by Patrao et al. was among the largest in real-world studies, the study featured worse baseline visual acuity than most other real-world studies (54.4 letters vs. 51.1–64.9 letters) [17–21, 24, 25].

Although we observed a similar median improvement in BCVA to figures reported in other real-world studies, relatively few eyes gained ≥15 letters (13.5%) in our study. For comparison, 21.5% of eyes in the OCEAN study and 17.1% of eyes in the POLARIS study gained ≥15 letters at 12 months [18, 19]. The percentage of eyes losing ≥15 letters was comparable with findings of other studies (6.8% vs. 6.3–7.2%) [18–20]. We also found that a greater improvement in vision (e.g., ≥10 letters) is unlikely for patients with baseline BCVA of >68 letters (approximate Snellen equivalent of ≥0.5). For this reason, these patients and treating physicians should have realistic expectations when initiating ranibizumab treatment. Although greater vision gains are experienced by those with worse baseline BCVA, their vision remains worse than of those with better baseline BCVA. In our study, this was observed throughout the entire follow-up period. The United Kingdom Diabetic Retinopathy Electronic Medical Record Users Group reported similar findings [17].

Aiming to represent everyday clinical practice, we did not exclude eyes based on BCVA, central subfield thickness, diabetes control, the duration of DME, or the number of injections. However, to avoid incorrect interpretation of changes in BCVA, we excluded eyes with advanced eye diseases (other than DR), significant media opacities, and intraocular surgery during the follow-up period. One of the reasons to exclude eyes with intraocular surgery was an improvement of vision after cataract surgery, which may lead to incorrect conclusions when analyzing overall results. Indeed, one recent real-world study of bevacizumab use for DME reported markedly greater vision gains among eyes with cataract surgery during the study period (mean BCVA change of +14 letters among eyes with on-study cataract surgery vs. +6 letters and +1 letter among eyes with prestudy and no cataract surgery, respectively) [27].

To our knowledge, this is the first real-world study on ranibizumab use for DME in Lithuania. As clinical practice varies to some extent among different countries, our study provides valuable insight into treatment patterns and vision outcomes achieved in a real-world clinical setting in Lithuania. One of the strengths of this study is that the loading dose was not required for the inclusion. This way, a more accurate picture of ranibizumab use in real-world clinical practice is provided, both in terms of treatment intensity and its efficacy. Some other real-world studies, however, did not include eyes without the loading phase completed [20, 22, 24].

A limitation of our study is that eyes treated with other anti-VEGF drugs were not included. At the time of the study, DME was treated almost exclusively with ranibizumab in our hospital because bevacizumab is not approved for ocular use and the cost of aflibercept treatment for DME was not covered by the national health insurance in Lithuania. Although this changed in September 2017, the follow-up duration at the time of data collection was not sufficient for the eyes receiving aflibercept treatment to be included in our study. By analyzing the eyes treated with ranibizumab only, we avoid the potential effect of differences among anti-VEGF drugs on the results of the study. In contrast, several other real-world studies analyzed eyes treated with different anti-VEGF agents [21, 24, 25, 28]. This complicates the comparison of outcomes if different anti-VEGF drugs are not analyzed separately.

## 5. Conclusions

Compared with randomized prospective clinical trials (PROTOCOL I, RESTORE, RISE, RIDE, RETAIN, REVEAL, and PROTOCOL T), patients in our study received less frequent injections and achieved lower vision gains [11–16]. The intensive treatment schedule used in clinical trials was not followed in a real-world clinical practice. Despite that, intravitreal ranibizumab for diabetic macular edema was still effective in a real-world clinical setting, with most eyes gaining or maintaining vision.

## Figures and Tables

**Table 1:** Baseline characteristics of patients and study eyes.

Characteristic	Result
Baseline characteristics of patients (*n* = 62)	
Gender: women – *n* (%)	30 (48.4%)
Age (years) – median (min; max)	59.5 (25; 79), *n* = 62
Patients with both eyes included in the study – *n* (%)	12 (19.4%)
Diabetes type – *n* (%)	
Type 1	13 (21.0%)
Type 2	49 (79.0%)
Duration of diabetes (full years) – median (min; max)	14 (0; 39), *n* = 59
HbA1c (%) – median (min; max)	7.9 (5.9; 10.3), *n* = 41

Baseline characteristics of study eyes (*n* = 74)	
BCVA (approxETDRS letters) – median (min; max)	62 (2; 85), *n* = 74
Snellen BCVA – median (min; max)	0.35 (0.01; 1), *n* = 74
Lens status: pseudophakic – *n* (%)^∗^	19 (25.7%)
Proliferative DR – *n* (%)	23 (31.1%)
Duration of DR (years) – median (min; max)	1.82 (0.01; 11.90), *n* = 71
Duration of DME (years) – median (min; max)	0.81 (0.00; 7.24), *n* = 72
Prior photocoagulation for DME – *n* (%)^∗∗^	40 (54.1%)
Sessions – median (min; max)	2 (1; 11), *n* = 40
Prior panretinal photocoagulation – *n* (%)^∗∗^	22 (29.7%)
Sessions – median (min; max)	2.5 (1; 9), *n* = 22
Prior anti-VEGF treatment – *n* (%)	11 (14.9%)
Injections – median (min; max)	4 (1; 7), *n* = 11
Prior intravitreal triamcinolone treatment – *n* (%)	10 (13.5%)
Injections – median (min; max)	1 (1; 2), *n* = 10

^∗^Unknown lens status - 3 eyes (4.1%),^∗∗^missing data regarding prior photocoagulation - 1 eye (1.4%). HbA1c, glycated hemoglobin; BCVA, best-corrected visual acuity; approxETDRS, approximate Early Treatment Diabetic Retinopathy Study letter score; DR, diabetic retinopathy; DME, diabetic macular edema; anti-VEGF, antivascular endothelial growth factor agents.

**Table 2:** BCVA outcomes among all eyes and within baseline BCVA subgroups.

Parameter	All eyes (*n* = 74)	Baseline BCVA subgroups		
		<55 approxETDRS letters (*n* = 26)	55–68 approxETDRS letters (*n* = 24)	>68 approxETDRS letters (*n* = 24)
BCVA at different time points (approxETDRS letters) – median (min; max)				
Baseline	62 (2; 85), *n* = 74	35 (2; 50), *n* = 26	62 (59; 67), *n* = 24	77 (70; 85), *n* = 24
365 ± 60 days after baseline	65 (2; 85)^∗^, *n* = 65	42 (2; 70), *n* = 24	65 (42; 85), *n* = 21	83 (62; 85), *n* = 20
730 ± 60 days after baseline	66 (2; 85)^∗∗^, *n* = 30	42 (2; 70), *n* = 10	67.5 (44; 85), *n* = 10	76 (51; 85), *n* = 10

Change in BCVA from baseline at different time points (approxETDRS letters) – median (min; max)				
365 ± 60 days after baseline	5 (−23; 45), *n* = 65	7 (−18; 45), *n* = 24	5 (−23; 26), *n* = 21	5 (−8; 8), *n* = 20
730 ± 60 days after baseline	5 (−34; 37), *n* = 30	7 (−8; 37), *n* = 10	5 (−15; 26), *n* = 10	2 (−34; 8), *n* = 10

Change in BCVA from baseline at the first visit ≥365 days after baseline – *n* (%)				
Gain of ≥15 letters	10 (13.5%)	9 (34.6%)	1 (4.2%)	0
Gain of 10–14 letters	6 (8.1%)	1 (3.8%)	5 (20.8%)	0
Gain of 5–9 letters	21 (28.4%)	5 (19.2%)	6 (25.0%)	10 (41.7%)
Gain/loss of ≤4 letters	18 (24.3%)	3 (11.5%)	6 (25.0%)	9 (37.5%)
Loss of 5–9 letters	13 (17.6%)	4 (15.4%)	4 (16.7%)	5 (20.8%)
Loss of 10–14 letters	1 (1.4%)	0	1 (4.2%)	0
Loss of ≥15 letters	5 (6.8%)	4 (15.4%)	1 (4.2%)	0

^∗^ *p* = 0.026; ^∗∗^*p* = 0.024. The Wilcoxon signed rank test used to compare with BCVA at baseline. BCVA, best-corrected visual acuity; approxETDRS, approximate Early Treatment Diabetic Retinopathy Study letter score.

## Data Availability

The datasets used and/or analyzed in this study are available from the corresponding author on reasonable request.
